# Do newly arrived migrants represent a COVID-19 burden? Data from the Italian information flow: Leuconoe Grazia Sisti

**DOI:** 10.1093/eurpub/ckac130.171

**Published:** 2022-10-25

**Authors:** LG Sisti, A Di Napoli, A Petrelli, A Diodati, A Cavani, C Mirisola, G Costanzo

**Affiliations:** National Institute for Health, Migration and Poverty, Rome, Italy

## Abstract

**Background:**

Migrants who reach host countries irregularly are often perceived as increasing the COVID-19 burden. Italy is a transit and destination country for migrants who cross the Central Mediterranean route. During the pandemic, all migrants who disembarked on the Italian shores have been COVID-19 tested and quarantined. To investigate the incidence of SARS-CoV-2 infection in this population, the INMP, together with the Italian Ministry of the Interior, set a specific information flow collecting data about the infection and possible outcomes.

**Methods:**

The observation period was from January 2021 to January 2022. COVID-19 tests used were molecular and antigenic. Positive cases detected both at the arrival and during the quarantine period, have been registered on an ad hoc INMP online platform. Migrants’ SARS-CoV-2 incidence rate (per 1,000) - with 95% CI - was therefore calculated. The Incidence Ratio (IR) was used to compare the migrants’ incidence rate with that of the resident population in Italy, in the same period and corresponding age group.

**Results:**

Among 70,512 migrants (91% males and 9% females, all <60years old) who landed in Italy during the observation period, 2,861 tested positive, with an incidence rate of 40.6 (39.1-42.1) cases per 1,000. In the same period, an incidence rate of 177.6 (177.5-177.8) has been recorded in the resident population, with an IR of 0.22 (0.22-0.23). 89.9% of cases were males and almost half (49.6%) belonged to the age group 25-39years old. 99% of cases reported no symptoms, no relevant comorbidity has been reported and no cases have been hospitalized.

**Conclusions:**

Our findings clearly highlight the low rate of SARS-CoV-2 infection in migrants reaching Italy by sea with an incidence rate that is roughly a quarter of that of the resident population, encouraging the opportunity to investigate the reasons for such an observation. Moreover, our study confirms the “healthy migrant effect” in migrants reaching Italy by sea.

**Key messages:**

- Irregular migrants arriving in Italy did not increase the COVID-19 burden in the country, thus alarmism is not justified.
- Further studies are needed to investigate the reasons for the lower incidence observed.

